# Acute-phase reactants as predictors of chronic kidney disease incidence in Africans: the population-based prospective RODAM cohort study

**DOI:** 10.1093/ije/dyag057

**Published:** 2026-05-01

**Authors:** Muhulo M Mungamba, Felix P Chilunga, Eva L Van Der Linden, Erik Buene, Charles F Hayfron-Benjamin, Karlijn A C Meeks, Samuel N Darko, Ellis Owusu-Dabo, Liffert Vogt, Bert-Jan Van Den Born, Benedicta N Nkeh-Chungag, Charles Agyemang

**Affiliations:** Department of Public and Occupational Health, Amsterdam Public Health Research institute, Amsterdam University Medical Centers, University of Amsterdam, Amsterdam, The Netherlands; Department of Human Biology, Faculty of Health Sciences, Nelson Mandela Drive, Walter Sisulu University, Mthatha, South Africa; Department of Public and Occupational Health, Amsterdam Public Health Research institute, Amsterdam University Medical Centers, University of Amsterdam, Amsterdam, The Netherlands; Department of Public and Occupational Health, Amsterdam Public Health Research institute, Amsterdam University Medical Centers, University of Amsterdam, Amsterdam, The Netherlands; Department of Public and Occupational Health, Amsterdam Public Health Research institute, Amsterdam University Medical Centers, University of Amsterdam, Amsterdam, The Netherlands; Department of Public and Occupational Health, Amsterdam Public Health Research institute, Amsterdam University Medical Centers, University of Amsterdam, Amsterdam, The Netherlands; Department of Vascular Medicine, Amsterdam Cardiovascular Sciences, Amsterdam University Medical Centers, University of Amsterdam, Amsterdam, The Netherlands; Department of Anaesthesia and Critical Care, University of Ghana Medical School/Korle Bu Teaching Hospital, Kumasi, Ghana; Department of Public and Occupational Health, Amsterdam Public Health Research institute, Amsterdam University Medical Centers, University of Amsterdam, Amsterdam, The Netherlands; Center for Research on Genomics and Global Health, National Human Genome Research Institute, National Institutes of Health, Bethesda, MD, United States; Division of Endocrinology, Diabetes and Nutrition, Department of Medicine, University of Maryland School of Medicine, Baltimore, MD, United States; Department of Molecular Medicine, School of Medicine and Dentistry, Kwame Nkrumah University of Science and Technology, Kumasi, Ghana; Department of Global and International Health, School of Public Health, Kwame Nkrumah University of Science and Technology, Kumasi, Ghana; Department of Internal Medicine, Section Nephrology, Amsterdam Cardiovascular Sciences, Amsterdam UMC, University of Amsterdam, Amsterdam, The Netherlands; Department of Public and Occupational Health, Amsterdam Public Health Research institute, Amsterdam University Medical Centers, University of Amsterdam, Amsterdam, The Netherlands; Department of Vascular Medicine, Amsterdam Cardiovascular Sciences, Amsterdam University Medical Centers, University of Amsterdam, Amsterdam, The Netherlands; Department of Biological and Environmental Sciences, Faculty of Natural Sciences, Nelson Mandela Drive, Walter Sisulu University, Mthatha, South Africa; Department of Public and Occupational Health, Amsterdam Public Health Research institute, Amsterdam University Medical Centers, University of Amsterdam, Amsterdam, The Netherlands; Division of Endocrinology, Diabetes, and Metabolism, Department of Medicine, Johns Hopkins University School of Medicine, Baltimore, MD, United States

**Keywords:** chronic kidney disease, chronic inflammation, iron overload, inflammatory markers, ferritin, C-reactive protein, sub-Saharan Africa

## Abstract

**Background:**

Chronic kidney disease (CKD) and chronic inflammation are highly prevalent in African populations, yet their relationship remains understudied. We examined the association between acute-phase reactants (C-reactive protein and ferritin) as markers of acute and chronic inflammation and the incidence of CKD 6 years later in a prospective Ghanaian population-based cohort.

**Methods:**

Data from the prospective Research on Obesity and Diabetes among African Migrants (RODAM-Pros) cohort were analysed and included participants living in rural and urban Ghana and Ghanaian migrants in the Netherlands. Acute-phase reactants were assessed between 2012 and 2015, while CKD incidence was assessed between 2019 and 2021 by using the race-free Chronic Kidney Disease Epidemiology Collaboration (CKD-EPI) 2021 equation. Robust Poisson regression models adjusted for potential confounders were used to assess associations. We explored interactions with age, sex, education, and geographical location, and stratified C-reactive protein (CRP) analyses by using established clinical cutoffs. The role of ferritin as an iron-storage marker was also evaluated.

**Results:**

Among 1435 participants, the baseline CRP was not associated with CKD incidence at follow-up [adjusted incidence rate ratio (aIRR) 1.02; 95% confidence interval (CI): 0.84–1.15]. Higher ferritin levels were associated with increased CKD risk (aIRR 3.53; 95% CI: 2.42–5.01) and albuminuria (aIRR 4.22; 95% CI: 2.87–6.10), but not with reduced estimated glomerular filtration rate (aIRR 0.99; 95% CI: 0.92–1.05). No effect modification was observed by age, sex, education, or geographical location. We found no evidence that iron overload or deficiency contributed to the ferritin–CKD relationship.

**Conclusion:**

Elevated ferritin levels, but not CRP levels, were associated with future CKD risk in Ghanaians. Multi-population prospective studies with repeated ferritin measurements are needed to better understand the links between ferritin, iron status, and CKD in African populations.

Key Messages

- We investigated whether inflammatory and iron-related markers [C-reactive protein (CRP) and ferritin] predict incident chronic kidney disease (CKD**)** over 6 years in a Ghanaian cohort.
- Elevated ferritin, but not CRP, was associated with higher CKD incidence and albuminuria; patterns also indicated that both iron deficiency and overload may contribute to early kidney dysfunction.
- Ferritin may serve as a more context-relevant biomarker of chronic inflammation and early CKD risk in African populations, supporting its use in targeted screening and iron-metabolism-based interventions.

## Introduction

Chronic kidney disease (CKD) is an escalating global public health issue [1, 2]. The CKD burden is particularly severe in populations from low- and middle-income regions such as sub-Saharan Africa (SSA). For instance, a 2014 systematic review found a CKD prevalence of 12% in urban areas and 17% in rural areas within SSA [3]. Our previous study comparing Ghanaians living in rural and urban settings in SSA and those who had migrated to Europe reported prevalence rates of 13%, 14%, and 10%, respectively [4]. Recent research in Uganda, South Africa, and Malawi revealed national prevalence rates ranging from 10% to 20% [5]. Additionally, we reported a CKD incidence of 12% in both rural and urban Ghanaian settings compared with 8% among Ghanaians residing in Europe over 6 years.

Understanding the factors contributing to this high CKD burden in SSA is crucial for the development of targeted prevention and treatment efforts. Classical risk factors such as unhealthy lifestyles, hypertension, diabetes, and genetics (e.g. *APOL1*) are thought to be the primary drivers of the CKD burden [6–8]. However, these factors do not fully explain the increased CKD incidence and prevalence in this region [9, 10].

Given the high prevalence of both acute and chronic infections, including human immunodeficiency virus (HIV), tuberculosis, hepatitis B and C, and malaria, in the SSA region [11, 12], there is growing evidence that these infections contribute to renal injury and increase the risk of CKD. For instance, HIV-associated nephropathy (HIVAN) is a well-documented cause of CKD [13], while hepatitis B and C infections have been linked to glomerulonephritis and other forms of renal disease [14, 15] and tuberculosis has been associated with immune-mediated renal damage [16]. The body’s inflammatory response to pathogens, particularly in the context of infectious diseases such as HIV, tuberculosis (TB), and hepatitis, can involve the influx of monocytes, proliferation of macrophages, and matrix expansion. This process contributes to glomerulosclerosis and tubulointerstitial injury—mechanisms that are also observed in diabetic nephropathy [17, 18]. However, infection-driven renal damage may involve distinct inflammatory pathways linked to the body’s immune response against pathogens. Additionally, inflammation can prompt glomerular cells to increase production and decrease the degradation of extracellular matrix proteins, contributing to glomerular hypertension, tubulointerstitial fibrosis, and renal scarring [6, 19–21].

While the role of inflammation in CKD has been extensively studied in high-income countries, where low-grade metabolic inflammation and autoimmune diseases are primary triggers, its role remains unclear in the SSA region, where infectious disease may play a significant role. Studies on inflammation and CKD in SSA have been limited in scope and sample size. For example, research in South Africa and Nigeria assessed inflammatory markers and CKD in just 40 and 67 adults, respectively [22, 23]. Another South African study focused solely on individuals with cardiovascular disease [24]. Additionally, a population-based study in Seychelles was cross-sectional, limiting the ability to establish temporality [25]. To date, no longitudinal studies have examined these associations. Moreover, no study has considered the impact of urbanization and international migration, which could influence the nature and extent of chronic inflammation, as rural populations may have lower inflammation related to obesity compared with urban and migrant populations [26].

To address this knowledge gap, we investigated the association between C-reactive protein (CRP) and ferritin as biomarkers of acute and chronic inflammation and CKD incidence over 6 years in a prospective Ghanaian population-based cohort. While numerous markers reflect inflammation, we focused on CRP and ferritin due to their availability within the prospective Research on Obesity and Diabetes among African Migrants (RODAM-Pros) cohort. We hypothesized that higher levels of both CRP and ferritin would be associated with an increased CKD risk, with effect modification by demographics such as age, sex, education, and geographical location (Supplementary Figure 1).

## Methods

### Study design and population

The study utilized data from the transcontinental population-based RODAM-Pros prospective cohort. Details have been published elsewhere [27]. Briefly, 4573 Ghanaian adults (≥18 years old) were enrolled between 2012 and 2015 across rural and urban sites in the Ashanti region of Ghana and in Amsterdam, the Netherlands. Participants were randomly selected from enumeration areas based on the 2010 Ghana census and municipal registers in Amsterdam using country-of-birth indicators.

Baseline data collection included questionnaires, anthropometry, and blood samples, following a standardized protocol across all sites. Follow-up took place between 2019 and 2021. The follow-up response rates were 63% in rural Ghana, 44% in urban Ghana, and 68% in Amsterdam. Of those followed up, physical examinations were completed by 91% (rural), 95% (urban), and 53% (Amsterdam) of the participants.

This analysis included participants without CKD at baseline. We excluded 409 individuals with symptoms of acute infection (fever, cough, sore throat, shortness of breath, runny nose); those receiving treatment for TB, HIV, or hepatitis C; and those on systemic antibacterial, antifungal, corticosteroid, or antiviral medications. After these exclusions, a total of 1435 participants were included in the final analysis. All participants provided written informed consent.

#### CKD

CKD was defined by using KDIGO criteria: an estimated glomerular filtration rate (eGFR) of <60 mL/min/1.73m^2^ (Stages 3a to 5) and/or an albumin–creatinine ratio (ACR) of ≥3 mg/mmol [28]. For individuals with eGFR within the normal to mildly decreased range (Stages 1 and 2), CKD was diagnosed by using albuminuria as an additional diagnostic criterion. Full details are provided in Supplementary Section 1.

### Inflammatory marker assessment and categorization

High-sensitivity CRP and ferritin were measured in heparin plasma by using particle-enhanced immunoturbidimetric assays on a Pentra 400 Chemistry Analyzer (HORIBA ABX). CRP was categorized as <10 vs ≥10 mg/L to distinguish metabolic from infectious inflammation and <1, 1–3, and ≥3 mg/L to reflect cardiovascular risk levels [29–31]. Ferritin was evaluated both as a continuous measure and by using WHO cutoffs: deficiency (<15 ng/mL), normal (15–200 ng/mL in men; 15–150 ng/mL in women), and overload (>200 ng/mL in men; >150 ng/mL in women) [32, 33]. Full details are provided in Supplementary Section 1.

### Other measurements

In brief, we collected a comprehensive set of measurements from the RODAM cohort, including environmental and lifestyle measurements. Full details are provided in Supplementary Section 1.

### Statistical analyses

R (Version 4.2.1) was utilized for the data analysis. Baseline characteristics were presented as mean ± standard deviation, median (interquartile range), or frequencies/percentages. Robust Poisson regression was used to assess the association between baseline CRP and ferritin (standardized as z-scores) and incident CKD at the 6-year follow-up. Models were adjusted for demographics, lifestyle, anthropometrics, and medication use. Interaction terms were tested for age, sex, education, location, and between CRP and ferritin.

CRP was also analysed by using clinical cutoffs: <10 vs ≥10 mg/L (to distinguish metabolic vs infectious inflammation) and <1, 1–3, and ≥3 mg/L (reflecting cardiovascular risk levels) [31–33]. Ferritin was analysed as a predictor of CKD to disentangle its role in inflammation vs iron storage.

We further examined the association of ferritin with albuminuria (ACR <3 vs ≥3 mg/mmol) and eGFR (<60 vs ≥60 mL/min/1.73m^2^). Incidence rate ratios (IRRs), 95% confidence intervals (CIs), and exact *P* values were reported.

### Sensitivity analysis

To assess the loss-to-follow-up bias, we compared the baseline characteristics of included vs excluded participants by using *t*-tests for continuous variables and chi-squared tests for categorical variables.

## Results

### Baseline characteristics of participants

**Figure 1:**
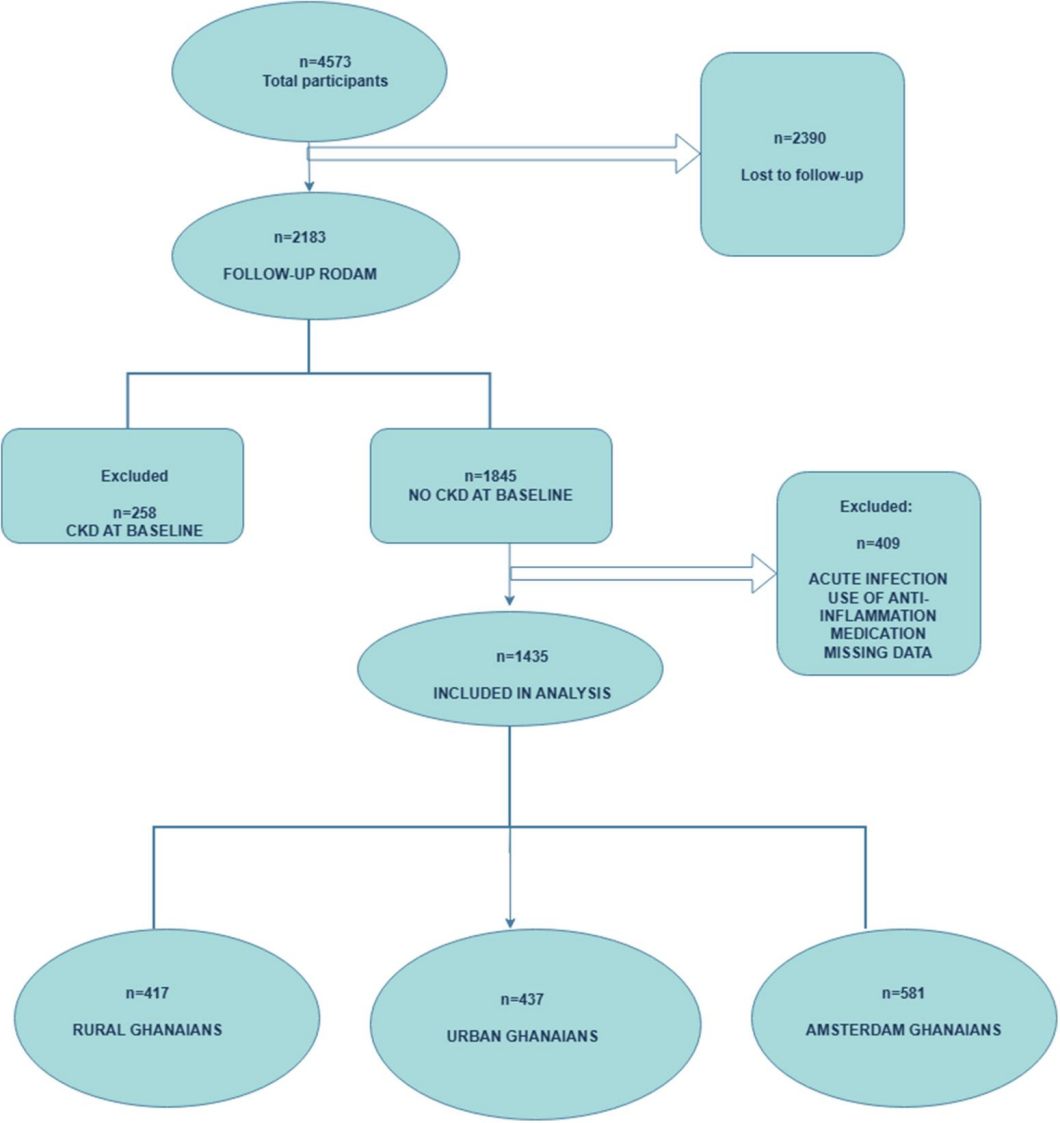
Flow diagram of study participants.

Of the 4573 RODAM participants enrolled (2012–15), 2390 (52%) were lost to follow-up. Among the 2183 remaining, 1845 had no CKD at baseline. After the exclusion of those with symptoms of acute infection or use of systemic anti-inflammatory, antibacterial, antifungal, or antiviral medication, 1435 participants were included: 417 (29.1%) from rural Ghana, 437 (30.5%) from urban Ghana, and 581 (40.5%) migrants in Amsterdam (Fig. 1).

Over 6 years, 159 developed CKD. Compared with the 1276 without CKD, cases were older (50 ± 11 vs 45 ± 11 years), more often female, and had higher body mass index (BMI), waist–hip ratios, and prevalences of hypertension (50.9% vs 36.1%) and diabetes (11.3% vs 5.7%). Albuminuria was more frequent (12.6% vs 5%) and the median eGFR was lower (80 vs 88 mL/min/1.73m^2^). CKD cases were also less likely to have tertiary education (20.1% vs 29.3%) or high physical activity (28.6% vs 40.2%), but more likely to smoke (12.6% vs 8.3%) or consume alcohol (45.9% vs 39.1%) (Table 1).

**Table 1:** Baseline characteristics of participants with and without incident CKD.

Variables	**CKD incidence vs no CKD**			
	Overall *N* = 1435	No CKD *N* = 1276	Incident CKD *N* = 159	*P* values
**Demographics**				
Ghanaians in rural Ghana [*n* (%)]	417 (29.1)	365 (28.6)	52 (32.7)	.029
Ghanaians in urban Ghana [*n* (%)]	437 (30.5)	379 (29.7)	58 (36.5)	
Ghanaians in Amsterdam [*n* (%)]	581 (40.5)	532 (41.7)	49 (30.8)	
Age (years) [mean (SD)]	46 (11)	45 (11)	50 (11)	<.001
Sex [*n* (%)]				
Females	899 (62.6)	788 (61.8)	111 (69.8)	.048
Males	536 (37.4)	488 (38.2)	48 (30.2)	
Education [*n* (%)]				
Lower vocational	521 (38.1)	464 (38.2)	57 (37.0)	.084
Intermediate	213 (15.6)	198 (16.3)	15 (9.7)	
Higher vocational	74 (5.4)	67 (5.5)	7 (4.5)	
Employment status [*n* (%)]				
Full-time	449 (35.2)	404 (35.5)	45 (32.4)	.3
Part-time	616 (48.2)	545 (47.9)	71 (51.1)	
Social benefits	86 (6.7)	78 (6.9)	8 (5.8)	
Retired	14 (1.1)	11 (1.0)	3 (2.2)	
Unable to work	77 (6.0)	69 (6.1)	8 (5.8)	
Student	18 (1.4)	18 (1.6)	0 (0.0)	
**Anthropometry information**				
BMI (kg/m^2^) [median (IQR)]	26.1 (22.4–29.9)	26.0 (22.3–29.8)	26.3 (22.9–30.2)	.3
Waist–hip ratio [median (IQR)]	0.90 (0.86–0.95)	0.90 (0.85–0.94)	0.92 (0.88–0.96)	<.001
**Lifestyle information**				
Any alcohol consumption [*n* (%)]	443 (37.5)	386 (37.0)	57 (41.9)	.3
Smoking [*n* (%)]				
Present	89 (6.6)	78 (6.5)	11 (7.1)	.4
Past	31 (2.3)	30 (2.5)	1 (0.6)	
Physical activity [*n* (%)]				
Moderate	212 (20.5)	185 (20.3)	27 (22.0)	.3
High	600 (58.0)	536 (58.8)	64 (52.0)	
**Laboratory information**				
Albuminuria [*n* (%)]	84 (5.9)	64 (5.0)	20 (12.6)	<.001
Triglycerides (mmol/L) [median (IQR)]	0.86 (0.63–1.15)	0.85 (0.63–1.13)	0.98 (0.72–1.32)	.002
Cholesterol (mmol/L) [median (IQR)]	4.85 (4.12–5.57)	4.84 (4.12–5.56)	4.92 (4.23–5.80)	.2
Uric acid (µmol/L) [median (IQR)]	293 (245–352)	292 (245–350)	298 (256–360)	.2
Urine albumin (mg/L) [median (IQR)]	0.70 (0.30–2.30)	0.70 (0.30–2.20)	0.90 (0.30–2.90)	.082
Urine creatinine (mmol/L) [median (IQR)]	10 (7–15)	10 (7–15)	9 (6–14)	.035
Albumin–creatinine ratio (mg/mmol) [median (IQR)]	4.0 (4.0–5.0)	4.0 (4.0–4.0)	4.0 (4.0–11.0)	<.001
eGFR, median (IQR)	87 (77–100)	88 (77–100)	80 (71–90)	<.001
**Underlying conditions**				
Hypertension [*n* (%)]	542 (37.8)	461 (36.1)	81 (50.9)	<.001
Diabetes [*n* (%)]	91 (6.3)	73 (5.7)	18 (11.3)	.006
Obesity [*n* (%)]	345 (24.1)	301 (23.6)	44 (27.7)	.3
**Use of medication for the underlying health conditions**				
Hypertension medication [*n* (%)]	204 (14.2)	177 (13.9)	27 (17.0)	.3
Diabetes medication [*n* (%)]	37 (2.6)	29 (2.3)	8 (5.0)	.057
**Markers of inflammation**				
CRP measurement (mg/L) [median (IQR)]	0.70 (0.30–2.30)	0.70 (0.30–2.20)	0.90 (0.30–2.90)	.082
Ferritin (ng/mL) [median (IQR)]	61 (29–104)	60 (29–102)	62 (33–126)	.050

Data are presented as percentages, means (standard deviations), or medians (interquartile ranges [IQR]). Percentages are rounded to one decimal place and may not sum to 100%. HDL, high-density lipoprotein; LDL, low-density lipoprotein; IQR, Interquartile range. *P* values were calculated by using the Wilcoxon rank-sum test for continuous variables and the Pearson chi-squared test or Fisher’s exact test for categorical variables.

**Figure 2:**
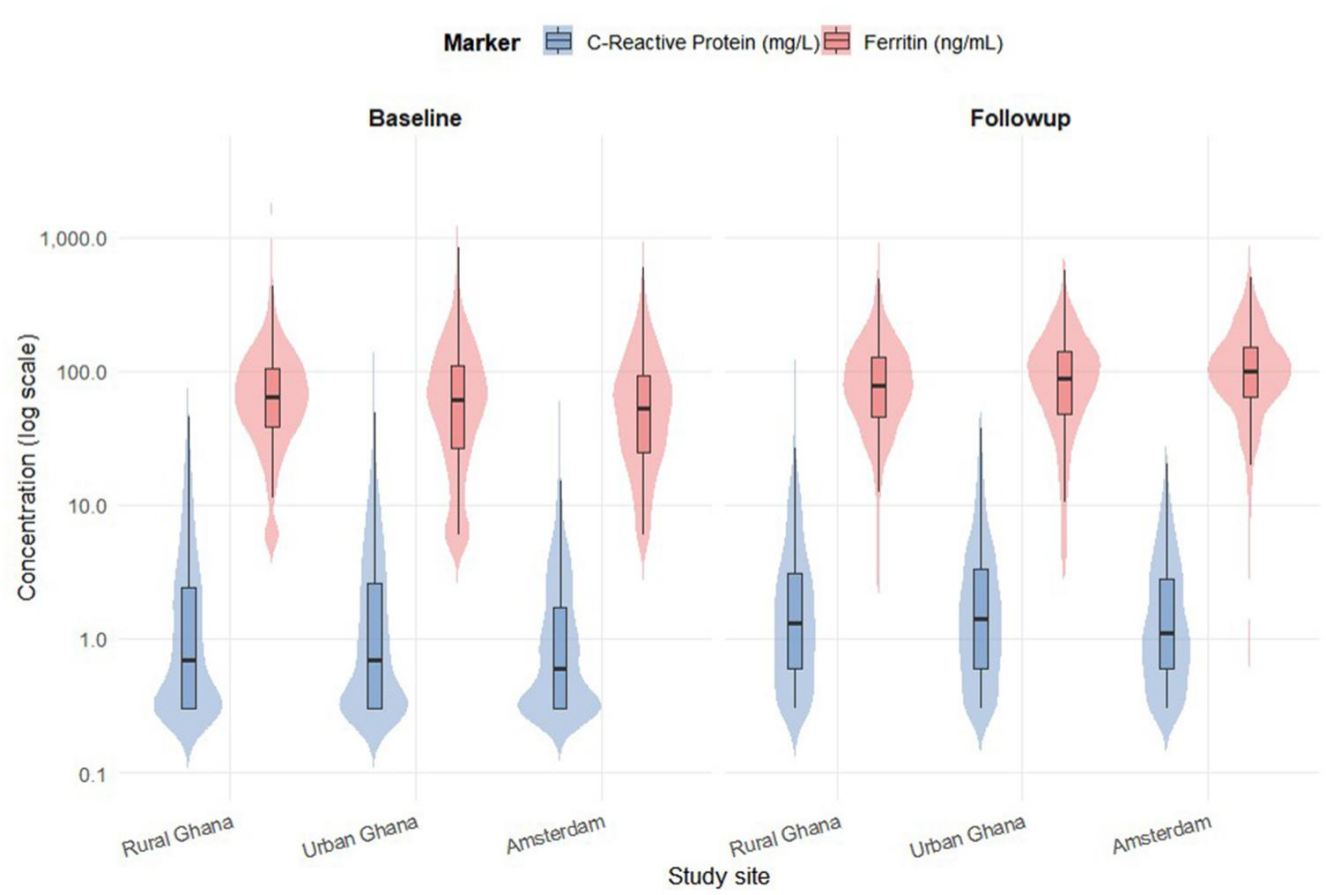
Distribution of inflammatory markers by study site.

The CRP concentrations were broadly similar across the sites, with rural Ghana at 0.70 mg/L (Interquartile range [IQR]: 0.30–2.40), 0.70 mg/L (IQR: 0.30–2.60) in urban Ghana, and 0.60 mg/L (IQR: 0.30–1.70) in Amsterdam. The proportion of participants with high CRP ranged from 21% to 27%. The ferritin levels showed site variation, with rural Ghana at 64.3 ng/mL (IQR: 38.5–103.9), 61.5 ng/mL (IQR: 26.5–109.8) in urban Ghana, and 53.1 ng/mL (IQR: 24.9–92.0) in Amsterdam. The follow-up ferritin levels remained the highest in Amsterdam (98.0 ng/mL, IQR: 61.0–149.0). The proportion with elevated ferritin also differed across the sites (Fig. 2 and Supplementary Table 1).

### Associations of CRP levels with CKD markers 6 years later

The z-standardized CRP levels were not associated with incident CKD based on KDIGO criteria [adjusted IRR (aIRR): 1.02, 95% CI: 0.84–1.15], albuminuria (aIRR: 0.99, 95% CI: 0.78–1.14), or decreased eGFR (aIRR: 0.99, 95% CI: 0.92–1.05) (Table 2). The CRP levels also showed no clear association with a change in eGFR over time (Supplementary Table 2).

**Table 2:** Association of inflammatory markers with CKD incidence, albuminuria, and eGFR of participants without CKD at the RODAM baseline.

Markers	Model 1 IRR (95% CI)	Model 2 IRR (95% CI)	Model 3 IRR (95% CI)	Model 4 IRR (95% CI)	Model 5 IRR (95% CI)
**CKD**					
CRP	1.10 (0.97–1.19)	1.06 (0.93–1.15)	1.04 (0.88–1.16)	1.03 (0.86–1.15)	1.02 (0.84–1.15)
Ferritin	**1.14 (1.02–1.23)**	**1.18 (1.05–1.28)**	**1.18 (1.04–1.28)**	**1.19 (1.04–1.32)**	**3.53 (2.42–5.01)**
**Albuminuria**					
CRP	1.02 (0.83–1.15)	0.98 (0.79–1.11)	1.01 (0.81–1.15)	1.01 (0.81–1.14)	0.99 (0.78–1.14)
Ferritin	1.11 (0.97–1.22)	**1.18 (1.03–1.29)**	**1.19 (1.05–1.30)**	**1.22 (1.06–1.38)**	**4.22 (2.87–6.10)**
**eGFR**					
CRP	0.98 (0.91–1.04)	0.98 (0.91–1.04)	0.99 (0.92–1.05)	0.99 (0.92–1.05)	0.99 (0.92–1.05)
Ferritin	0.99 (0.93–1.04)	0.99 (0.93–1.05)	1.01 (0.93–1.06)	1.01 (0.93–1.06)	1.01 (0.80–1.24)

Results are based on robust Poisson regression models. The predictors were CRP and ferritin, and the outcomes were CKD, albuminuria, and eGFR. CKD was defined based on the race-free Chronic Kidney Disease Epidemiology Collaboration (CKD-EPI) 2021 equation. Albuminuria was categorized as ACR of <3 and ≥3 mg/mmol. eGFR was categorized as <60 and ≥60 mL/min/1.73 m^2^. CRP and ferritin were standardized as z-scores. All continuous variables were standardized as z-scores. Model 1: unadjusted. Model 2: adjusted for age and sex. Model 3: Model 2 plus education. Model 4: Model 3 plus BMI, smoking, physical activity, alcohol consumption, obesity, diabetes, hypertension, and hypertension and diabetes treatment. Model 5: Model 4 with additional adjustment for follow-up CRP or ferritin.

Categorical analyses of CRP levels, including <10 vs ≥10 mg/L and <1, 1–3, and >3 mg/L, also did not reveal robust associations with CKD incidence (aIRR for CRP ≥0 mg/L: 1.46, 95% CI: 0.73–2.63; CRP 1–3 mg/L: 1.15, 95% CI: 0.74–1.76; CRP >3 mg/L: 1.09, 95% CI: 0.66–1.75, compared with CRP <1 mg/L) (Table 3).

**Table 3:** Association of CRP at different category levels with incident CKD 6 years later.

CRP levels	*N*	Model 1 IRR (95% CI)	Model 2 IRR (95% CI)	Model 3 IRR (95% CI)	Model 4 IRR (95% CI)	Model 5 IRR (95% CI)
**CRP <10 and ≥10 mg/L**						
<10	1252	1.00 (Reference)	1.00 (Reference)	1.00 (Reference)	1.00 (Reference)	1.00 (Reference)
≥10	68	1.80 (0.97–3.06)	1.62 (0.87–2.76)	1.61 (0.84–2.79)	1.65 (0.85–2.92)	1.46 (0.73–2.63)
**CRP <1, 1–3, and >3 mg/L**						
<1	757	1.00 (Reference)	1.00 (Reference)	1.00 (Reference)	1.00 (Reference)	1.00 (Reference)
1–3	269	1.28 (0.85–1.90)	1.12 (0.74–1.66)	1.14 (0.74–1.70)	1.25 (0.80–1.94)	1.15 (0.74–1.76)
>3	262	1.34 (0.89–1.99)	1.20 (0.79–1.79)	1.23 (0.80–1.85)	1.17 (0.72–1.86)	1.09 (0.66–1.75)

Results are based on robust Poisson regression models. The predictor was CRP and the outcome was CKD. CKD was defined based on the race-free Chronic Kidney Disease Epidemiology Collaboration (CKD-EPI) 2021 equation. Model 1: unadjusted. Model 2: adjusted for age and sex. Model 3: Model 2 plus education. Model 4: Model 3 plus smoking, physical activity, alcohol consumption, obesity, diabetes, study site, and hypertension. Model 5: Model 4 with additional adjustment for follow-up CRP.

No evidence of effect modification was found for the CRP–CKD association by age (IRR: 1.01, 95% CI: 0.99–1.01), sex (IRR: 1.02, 95% CI: 0.98–1.11), education (IRR: 0.83, 95% CI: 0.20–1.28), or location (IRR: 0.94, 95% CI: 0.74–1.06) (Supplementary Table 3).

### Association of ferritin levels with CKD markers 6 years later

A 1-SD increase in ferritin was associated with a higher risk of incident CKD (aIRR: 3.53, 95% CI: 2.42–5.01) and albuminuria (aIRR: 4.22, 95% CI: 2.87–6.10), but not with decreased eGFR (aIRR: 1.01, 95% CI: 0.80–1.24) (Table 2). Ferritin also showed no clear association with change in eGFR (Supplementary Table 2).

No interactions were observed between ferritin and CRP (IRR: 0.99, 95% CI: 0.99–1.01), age (IRR: 1.01, 95% CI: 0.99–1.01), sex (IRR: 1.01, 95% CI: 0.99–1.02), education (IRR: 1.01, 95% CI: 0.99–1.01), or geographical location (IRR: 0.99, 95% CI: 0.99–1.01) (Table 4).

**Table 4:** Interaction between baseline ferritin and CRP and demographic factors in relation to CKD.

Markers	IRR (95% CI)	*P* values
**CRP**		
Ferritin * CRP	0.99 (0.99–1.01)	.294
**Age**		
Ferritin * Age	1.01 (0.99–1.01)	.614
**Sex**		
Ferritin * Males	1.00 (Reference)	1.00 (Reference)
Ferritin * Females	1.01 (0.99–1.02)	.816
**Education**		
Ferritin * No education	1.00 (Reference)	1.00 (Reference)
Ferritin * Low education	1.01 (0.99–1.01)	.412
Ferritin * Intermediate education	0.99 (0.97–1.01)	.216
Ferritin * High education	1.01 (0.99–1.01)	.131
**Geographical location**		
Ferritin * Rural Ghanaians	1.00 (Reference)	1.00 (Reference)
Ferritin * Urban Ghanaians	0.99 (0.99–1.01)	.763
Ferritin * Amsterdam Ghanaians	0.99 (0.99–1.01)	.673

Results are based on robust Poisson regression models assessing interaction terms between ferritin and selected variables. The outcome was CKD. CKD was defined based on the race-free Chronic Kidney Disease Epidemiology Collaboration (CKD-EPI) 2021 equation. Reference categories were male sex, no education, and rural Ghana.

Ferritin serves as both an inflammatory marker and an indicator of iron storage. When ferritin was categorized by iron status (deficiency, normal, overload), no associations were observed with CKD incidence, albuminuria, or eGFR, although a trend was noted toward higher albuminuria and lower eGFR in those with iron overload (Supplementary Table 4).

### Sensitivity analysis

Overall, most characteristics, including kidney-related markers, were similar between the included participants and those lost to follow-up. However, the follow-up loss was lower in rural Ghana and those lost had slightly higher ACR and lower median eGFR (86 vs 87 mL/min/1.73m^2^). They also had higher rates of hypertension, diabetes, and diabetes medication use (Supplementary Table 5).

## Discussion

### Key findings

We aimed to investigate the association between inflammatory markers (CRP and ferritin) with CKD incidence over 6 years. Ferritin emerged as a stronger predictor than CRP, showing positive associations with CKD (KDIGO criteria) and albuminuria, but not with eGFR. CRP showed no clear association with CKD, although estimates suggested a trend toward increased risk. The associations were not modified by age, sex, migration status, education, or iron status.

### Discussion of key findings

While CRP has been identified as a risk marker for CKD in high-income countries, our findings did not show an association between CRP levels and CKD incidence over a 6-year period. This is surprising considering that CRP has been reported cross-sectionally to be associated with CKD risk in some small studies in South Africa, Nigeria, Ghana, and Seychelles [22–25], though the temporality was uncertain. CRP is a well-established biomarker of systemic inflammation [34, 35]. Elevated CRP levels in the absence of acute infection typically indicate chronic low-grade inflammation, which is a known contributor to endothelial dysfunction, oxidative stress, and glomerular injury, ultimately promoting kidney-function decline [34, 36–39]. The absence of an observed association in our cohort may suggest an alternative mechanism such as inflammatory adaptation. In populations with prolonged exposure to infectious or environmental antigens, the immune system may recalibrate to a higher CRP “set point” without causing proportional tissue injury. In such contexts, elevated CRP levels may reflect generalized immune activation rather than pathologic inflammation. Supporting this notion, a study in Tanzania found that urban living was associated with increased inflammatory markers among otherwise healthy individuals, indicating immune adaptation to environmental and metabolic stressors rather than disease-related inflammation [40].

Our study revealed a positive association between elevated ferritin levels and an increased risk of CKD incidence over a 6-year period, with 1 SD in the ferritin levels associated with a 3.53-fold higher risk of CKD. This association between elevated ferritin levels and CKD risk is particularly noteworthy, as it supports the role of ferritin as a marker of chronic inflammation in this population. Ferritin—an acute-phase reactant and marker of iron storage—reflects chronic inflammation, which is known to be involved in CKD pathogenesis [41, 42]. This finding is consistent with those of studies on other populations that have linked higher ferritin levels with CKD progression [41, 43]. However, few cross-sectional studies have specifically examined ferritin in relation to CKD across different ancestral populations. For instance, one study in Nigeria found that higher ferritin levels were associated with worse renal outcomes in CKD patients, supporting our findings [44]. Another study in South Africa also reported higher ferritin levels being associated with CKD risk in patients, similarly to our finding [45]. Although these studies were conducted in clinical settings with small sample sizes, the consistency in findings highlights the need to consider ferritin as a potential predictor of future CKD and warrants further research to elucidate the mechanisms by which ferritin might contribute to CKD. It is possible that elevated ferritin, as a marker of iron overload, may contribute to kidney damage through mechanisms involving oxidative stress and inflammation, and further studies are needed to clarify these pathways.

We found no evidence of interactions between ferritin levels and age, female sex, higher education, or migration status. This suggests that the relationship between elevated ferritin and CKD risk does not differ across these subgroups. Our initial hypothesis proposed that higher CRP and ferritin levels would be associated with increased CKD risk, with potential effect modification by demographics such as age, sex, education, and migration status. However, our findings do not support interaction with these variables, which strengthens confidence in the robustness of the observed association. While ferritin may reflect inflammation, it might also have a direct impact on CKD development through mechanisms such as oxidative stress, which can damage kidney cells regardless of the demographic factors. Thus, the role of ferritin could be dual, as both a marker of inflammation and a contributor to CKD pathology. The lack of effect modification by demographic factors in the ferritin–CKD relationship suggests that the impact of elevated ferritin might be universal. This consistency across subgroups could simplify risk stratification and guide more targeted intervention strategies in clinical practice.

Ferritin is often used as a marker of inflammation in studies exploring CKD risk, but it also serves as an indicator of iron status, with high levels indicating iron overload and low levels indicating iron deficiency. Although our study did not find clear evidence of an association with CKD risk over 6 years based on ferritin categories, we did observe trends suggesting potential clinical relevance. Both iron deficiency and iron overload were associated with higher rates of albuminuria and decreased eGFR, although these trends were not precise and may reflect the limited sample size. These observations suggest that the relationship between ferritin levels and CKD might be partly explained by iron status. Iron-metabolism disorders are known to impact kidney function through mechanisms involving oxidative stress and inflammation. Our findings align with those of studies in South Africa and Nigeria showing higher rates of iron deficiency in patients with decreased eGFR [32, 33]. While we did not find an association between iron status and CKD risk, the trends we observed point towards a possible role of both iron overload and iron deficiency in CKD progression. These trends warrant further investigation with larger cohorts or longer follow-up to better understand these relationships in African populations.

The findings of our study hold important clinical and public health implications for managing CKD risk in African populations. Elevated ferritin levels were linked to increased CKD incidence, suggesting ferritin as a potential biomarker for early detection and risk assessment. To better determine whether ferritin can reliably predict CKD risk, it is crucial to conduct detailed studies with multiple ferritin measurements, as well as information on acute infection and hemoglobin over time in relation to eGFR and albuminuria, and in multiple ethnic groups.

### Strengths and limitations

Key strengths include the longitudinal design, which enabled the assessment of CKD incidence and temporality over 6 years, and the inclusion of participants from rural Ghana, urban Ghana, and Ghanaian migrants in Amsterdam, capturing different geographical and environmental contexts within a relatively homogeneous population. This design represented the range of settings in which Africans may find themselves. We also assessed two distinct inflammatory markers (CRP and ferritin) and iron status, with adjustment for major demographic and lifestyle confounders, strengthening internal validity.

Limitations include the variation in response rates, with especially low follow-up in urban Ghana and Amsterdam, largely due to the COVID-19 pandemic. This may have introduced bias, although nonresponse analysis showed comparable baseline eGFR levels between included and excluded participants. We found no evidence of effect modification by location, but the findings may not be generalizable to other SSA or migrant populations given the differing genetic, environmental, and healthcare factors. We lacked detailed data on *APOL1* variants and long-term herbal medicine use—important CKD risk factors in SSA that were unavailable. Participants on treatment for chronic infectious diseases (e.g. HIV, TB) were excluded. However, undiagnosed or untreated chronic infections could still have influenced the biomarker levels. eGFR was measured only twice, so some CKD cases may reflect acute impairment. The absence of *iohexol* or cystatin-C data, recommended for more accurate kidney-function assessment, is another limitation. Finally, CRP and ferritin are nonspecific inflammation markers; future research should incorporate a broader biomarker panel.

## Conclusion

Elevated ferritin levels, but not CRP levels, were associated with future CKD risk in Ghanaians. Multi-population prospective studies with repeated ferritin measurements are needed to better understand the links between ferritin, iron status, and CKD in African populations.

## Ethics approval

Ethical approval was obtained from the Committee on Human Research, Publication & Ethics in Ghana (CHRPE/AP/172/19) and the Institutional Review Board of the AMC, University of Amsterdam (NL32251.018.10). All participants provided written informed consent.

## Supplementary Material

dyag057_Supplementary_Data

## Data Availability

The data that support the findings of this study are available from the corresponding author upon reasonable request. Due to privacy and ethical considerations, the data are not publicly available. Requests for access to the data should be directed to the corresponding author and will be considered by the research RODAM study’s ethics committee. The data are stored in a secure database and can be accessed under the conditions set by the ethical guidelines of the RODAM study and the participating institutions.
