# Supplementary material for: Acute-phase reactants as predictors of chronic kidney disease incidence in Africans: the population-based prospective RODAM cohort study
Source: Int J Epidemiol. 2026 May 1;55(3):dyag057. doi: 10.1093/ije/dyag057 (PMC13132656; doi:10.1093/ije/dyag057)
Supplement: dyag057_Supplementary_Data [file dyag057_supplementary_data.docx]

**Supplementary material**

**Manuscript title:** Acute Phase Reactants as Predictors of Chronic Kidney Disease Incidence in Africans: The Population-Based Prospective RODAM Cohort Study

**Contents**

- **Supplementary Figure S1**. Conceptual framework of potential factors influencing chronic kidney disease
- **Supplementary Section S1**. Detailed description of measurements
- **Supplementary Table S1**. Distribution of ferritin and C-reactive protein (CRP) by study site
- **Supplementary Table S2**. Association of inflammatory markers with change in estimated glomerular filtration rate (ΔeGFR)
- **Supplementary Table S3**. Interaction between baseline C-reactive protein (CRP) and demographic factors in relation to chronic kidney disease (CKD)
- **Supplementary Table S4.** Association between iron status and chronic kidney disease (CKD) markers
- **Supplementary Table S5**. Baseline characteristics of included participants versus those lost to follow-up

**Supplementary Figure S1:** conceptualization of how potential factors may interplay.


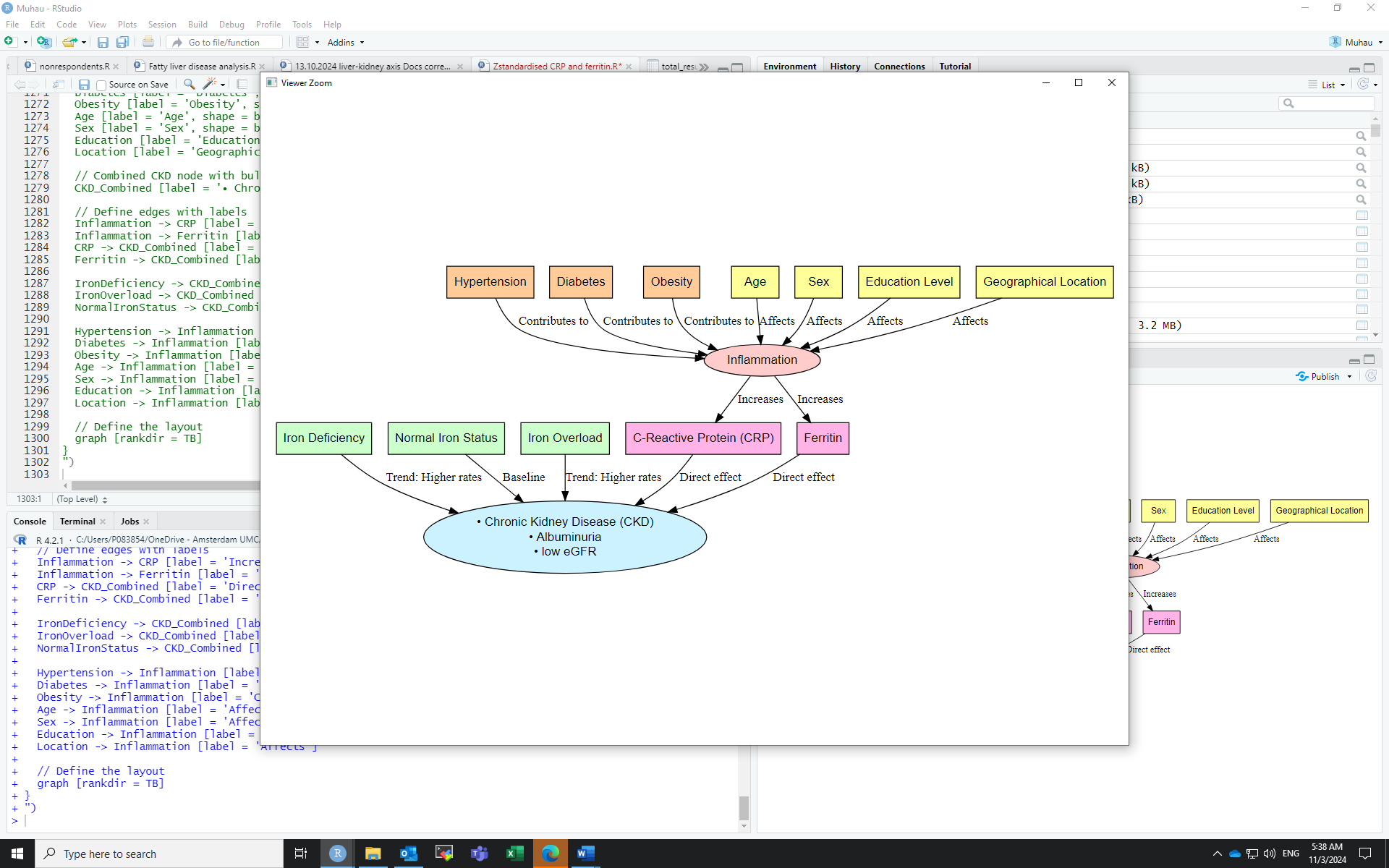


**Supplementary Section S1: Detailed account of measurements**

**Chronic kidney disease**

Participants provided early morning urine samples to analyse albumin and creatinine. Urinary-albumin-concentration (in mg/L) was determined using an immunochemical-turbidimetric-method and urinary-creatinine-concentration (in mmol/L) using kinetic spectrophotometric method (Roche Diagnostics). Albuminuria categories were based on urine albumin-to-creatinine ratio (ACR): A1 (<3 mg/mmol; normal to mildly increased), A2 (3-30 mg/mmol; moderately increased) and A3 (>30 mg/mmol; severely increased) (1).

Estimated glomerular filtration rate (eGFR) was calculated using the race-free CKD-EPI 2021 equation (2), selected for its improved accuracy in African populations and due to the absence of Iohexol-based measurements (3). Serum creatinine concentration (mmol/L) was determined using a kinetic colorimetric spectrophotometric isotope dilution mass spectrometry calibrated method (Roche Diagnostics).

CKD was defined using KDIGO criteria : an eGFR below 60 mL/min/1.73 m² (stages 3a to 5) and/or ACR of ≥3 mg/mmol (4). For individuals with eGFR in the normal to mildly decreased range (stages 1 and 2), CKD was diagnosed using albuminuria (i.e., ACR ≥ 3 mg/mmol) as an additional diagnostic criterion, indicating kidney damage despite preserved kidney function. Therefore, individuals with albuminuria and a normal eGFR were considered to have CKD.

**Inflammatory marker assessment and categorization**

**CRP measurement and categorization:** High-sensitivity CRP (hs-CRP) was measured in heparin plasma using a particle-enhanced immunoturbidimetric assay with a Pentra 400 Chemistry Analyzer (HORIBA ABX). CRP values were categorized as <10 mg/L and ≥10 mg/L to differentiate metabolic from potential infectious inflammation. Additional categories (<1, 1–3, and ≥3 mg/L) reflected low, average, and high cardiovascular risk levels (5-7).

**Ferritin measurement and Iron status categorization:** Ferritin levels were measured in heparin plasma using a particle-enhanced immunoturbidimetric assay. In this method, human ferritin agglutinates with latex particles coated with monoclonal anti-ferritin antibodies, and the resulting aggregates were measured turbidimetrically using a Pentra 400 Chemistry Analyzer (HORIBA ABX). Iron status was assessed using plasma ferritin levels, as recommended by the WHO: iron deficiency (ferritin <15 ng/mL), normal (ferritin >15 ng/mL to <200 ng/mL for males and >15 ng/mL to <150 ng/mL for females), and iron overload (ferritin >200 ng/mL for males, >150 ng/mL for females) (8, 9).

**Other measurements**

In addition to measurements related to chronic kidney disease (CKD) and chronic inflammation, we collected a comprehensive set of measurements from the RODAM cohort for analysis. These include age, sex, education levels, tobacco smoking, dietary patterns, alcohol consumption, physical activity, anthropometrics, blood pressure, fasting blood glucose, hypertension, diabetes, and use of medications.

Age was recorded in years; and sex was categorized into male and female. Educational level was assessed based on the highest education attained, with categories including never been to school, lower vocational, intermediate vocational, and higher vocational. Smoking status was categorized as yes, no, or ex-smoker, while alcohol use in the last 12 months was categorized as yes or no. The WHO steps questionnaire was used to derive physical activity in metabolic equivalent (MET, hours/week), which included physical activity at work, while commuting, and in leisure time. Answers were subsequently classified based on the guidelines of The IPAQ group into three levels of total physical activity (low, moderate, high) (10-12).

Body weight was measured in light clothing without shoes, using SECA 877 scales with a precision of 0.1 kg. Height was measured without shoes using a portable stadiometer (SECA 217) with a precision of 0.1 cm. Body mass index (BMI) was calculated as weight divided by height squared (kg/m^2^). Overweight was defined as a BMI of between 25 and 30 kg/m^2^, while obesity was defined as a BMI of 30 kg/m^2^ or higher. Waist circumference was measured in centimeters at the midpoint between the lower rib and the upper margin of the iliac crest. All these anthropometric measures were taken twice, and the mean was used for analysis.

Blood pressure (BP) was measured three times while participants were in a seated position after resting for at least 5 minutes. The measurements were taken using the Microlife WatchBP Home, a validated semi-automated device. The mean of the last two BP measurements was used for analysis. Hypertension was defined as systolic BP of 140 mmHg or higher and/or diastolic BP of 90 mmHg or higher, or the use of antihypertensive medication, per WHO criteria (11).

Fasting plasma glucose concentration was determined using the enzymatic hexokinase method. Type 2 diabetes was defined based on the diagnostic criteria set by the WHO, including a fasting glucose level of 7.0 mmol/L or higher, current use of medication prescribed for diabetes treatment, or self-reported physician-diagnosed diabetes (13, 14). Serum uric acid concentration was measured using an enzymatic method (Trinder) in µmol/L. The concentration of total cholesterol was assessed using colorimetric test kits.

Medication use was assessed by asking participants to bring their prescribed medications to the physical exam and these were subsequently coded and categorized. For hypertension, antihypertensive medications included were diuretics, beta blockers, angiotensin converting enzyme (ACE) inhibitors, or Angiotensin Receptor Blockers (ARBs), either individually or in combination. For diabetes, hypoglycemic medications were considered including Metformin, Sulfonylureas, and Insulin.

**References**

1. Lameire NH, Levin A, Kellum JA, Cheung M, Jadoul M, Winkelmayer WC, et al. Harmonizing acute and chronic kidney disease definition and classification: report of a Kidney Disease: Improving Global Outcomes (KDIGO) Consensus Conference. Kidney international. 2021;100(3):516-26.

2. Miller WG, Kaufman HW, Levey AS, Straseski JA, Wilhelms KW, Yu HY, et al. National Kidney Foundation Laboratory Engagement Working Group recommendations for implementing the CKD-EPI 2021 race-free equations for estimated glomerular filtration rate: practical guidance for clinical laboratories. Clinical chemistry. 2022;68(4):511-20.

3. Fabian J, Kalyesubula R, Mkandawire J, Hansen CH, Nitsch D, Musenge E, et al. Measurement of kidney function in Malawi, South Africa, and Uganda: a multicentre cohort study. The Lancet Global Health. 2022;10(8):e1159-e69.

4. Levey AS, Eckardt K-U, Dorman NM, Christiansen SL, Hoorn EJ, Ingelfinger JR, et al. Nomenclature for kidney function and disease: report of a Kidney Disease: Improving Global Outcomes (KDIGO) Consensus Conference. Kidney international. 2020;97(6):1117-29.

5. DO SHISHEHBOR MH, Bhatt DL. Using C-reactive protein to assess cardiovascular disease risk. Cleveland clinic journal of medicine. 2003;70(7):635.

6. Lolekha PH, Chittamma A, Roberts WL, Sritara P, Cheepudomwit S, Suriyawongpaisal P. Comparative study of two automated high-sensitivity C-reactive protein methods in a large population. Clinical biochemistry. 2005;38(1):31-5.

7. Pearson TA, Mensah GA, Alexander RW, Anderson JL, Cannon III RO, Criqui M, et al. Markers of inflammation and cardiovascular disease: application to clinical and public health practice: a statement for healthcare professionals from the Centers for Disease Control and Prevention and the American Heart Association. circulation. 2003;107(3):499-511.

8. WHO C. Assessing the Iron Status of Populations. Geneva. World Health Organization. 2007.

9. Organization WH. WHO guideline on use of ferritin concentrations to assess iron status in populations: World Health Organization; 2020.

10. Armstrong T, Bull F. Development of the world health organization global physical activity questionnaire (GPAQ). Journal of Public Health. 2006;14:66-70.

11. Organization WH. Guideline for the pharmacological treatment of hypertension in adults: World Health Organization; 2021.

12. Osei TB, van Dijk A-M, Dingerink S, Chilunga FP, Beune E, Meeks KAC, et al. Reduced RANK Regression-Derived dietary patterns related to the fatty liver index and associations with type 2 diabetes mellitus among Ghanaian populations under transition: the RODAM study. Nutrients. 2021;13(11):3679.

13. Roth GA, Abate D, Abate KH, Abay SM, Abbafati C, Abbasi N, et al. Global, regional, and national age-sex-specific mortality for 282 causes of death in 195 countries and territories, 1980–2017: a systematic analysis for the Global Burden of Disease Study 2017. The Lancet. 2018;392(10159):1736-88.

14. Committee ADAPP, Committee: ADAPP. 2. Classification and diagnosis of diabetes: Standards of Medical Care in Diabetes—2022. Diabetes care. 2022;45(Supplement_1):S17-S38.

**Supplementary Table S1:** Distribution of ferritin and C-reactive protein (CRP) by study site (rural Ghana, urban Ghana, and Amsterdam)

| **Marker** | **Category** | **Overall** | **Rural Ghana** | **Urban Ghana** | **Amsterdam** | **p-value** |
| --- | --- | --- | --- | --- | --- | --- |
| **Ferritin, ng/mL** | Baseline, Median(IQR) | 60.50 (28.90-103.60) | 64.30 (38.50-103.90) | 61.50 (26.50-109.80) | 53.10 (24.90-92.00) | 0.004 |
|  | Follow-up, Median(IQR) | 82.22 (40.96-133.43) | 70.61 (33.41-120.56) | 82.41 (36.25-132.64) | 98.03 (61.00-149.00) | <0.001 |
|  | Change, n(%) | 20.84 (46.34) | 5.74 (41.69) | 16.10 (38.49) | 45.67 (50.98) | <0.001 |
|  | High, n(%) | 172 (8.8%) | 43 (5.5%) | 68 (8.2%) | 61 (10.7%) | 0.014 |
|  | Low, n(%) | 1,019 (91.2%) | 374 (94.5%) | 369 (91.8%) | 276 (89.3%) |  |
| **C-Reactive Protein**  **mg/L** | Baseline, Median(IQR) | 0.70 (0.30-2.30) | 0.70 (0.30-2.40) | 0.70 (0.30-2.60) | 0.60 (0.30-1.70) | 0.088 |
|  | Follow-up, Median(IQR) | 1.20 (0.60-3.00) | 1.30 (0.60-3.10) | 1.40 (0.60-3.30) | 1.10 (0.60-2.80) | 0.184 |
|  | Change, n(%) | 0.43 (9.44) | 0.61 (12.06) | 0.02 (9.66) | 0.72 (3.77) | 0.676 |
|  | High, n(%) | 294 (24.7%) | 108 (25.9%) | 116 (26.5%) | 70 (20.8%) | 0.141 |
|  | Low, n(%) | 897 (75.3%) | 309 (74.1%) | 321 (73.5%) | 267 (79.2%) |  |

*Baseline and follow-up values of serum ferritin and high-sensitivity CRP (hsCRP) are presented as median [interquartile range]; changes are shown as mean (standard deviation). High and low categories indicate the proportion of participants with elevated biomarker levels across sites. High CRP was defined as >3 mg/L, following American Heart Association and CDC recommendations. Elevated ferritin was defined as >200 ng/mL in men and >150 ng/mL in women, according to WHO population-level guidance (WHO 2020). P-values were derived from Kruskal-Wallis tests for non-normally distributed variables, ANOVA for mean differences across sites, and Chi-square tests for categorical comparisons.*

**Supplementary Table S2**: Association of inflammatory markers with delta eGFR of participants without CKD at the RODAM baseline.

| **Markers** | **Model 1**  **IRR(95% CI)** | **Model 2**  **IRR(95% CI)** | **Model 3**  **IRR(95% CI)** | **Model 4**  **IRR(95% CI)** | **Model 5**  **IRR(95% CI)** |
| --- | --- | --- | --- | --- | --- |
| **Delta eGFR** |  |  |  |  |  |
| C-Reactive protein | -0.12 (-0.98-0.73) | -0.18 (-1.03-0.66) | 0.21 (-0.71-1.14) | 0.07 (-0.87-1.02) | 0.09 (-0.86-1.05) |
| Ferritin | -1.27 (-2.12-0.41) | -0.62 (-1.52-0.27) | -0.46 (-1.40-0.48) | -0.72 (-1.73-0.27) | 3.85 (0.66-7.04) |

*Results based linear regression. Predictor =CRP or Ferritin, Outcome= delta eGFR. Delta eGFR is the change between baseline and follow-up eGFR. CRP and ferritin are converted to standardized z scores. All continuous determinants are also converted to standardized z-scores.* ***Model 1****= unadjusted.* ***Model 2*** *= adjusted for age and sex.* ***Model 3*** *= model 2 + education.* ***Model 4****= Model 3 + BMI, smoking, physical activity, alcohol consumption, obesity, diabetes, hypertension, hypertension and diabetes treatment. Model 5 = Model 4 with additional adjustment for follow-up CRP/Ferritin.*

**Supplementary Table S3**: Interaction between baseline CRP and demographic factors in the relationship with CKD

| **CRP levels** | **IRR (95% CI)** | **p-values** |
| --- | --- | --- |
| **Age** | | |
| CRP * Age | 0.99 (0.99-1.01) | 0.387 |
| **Sex** | | |
| CRP * Males | 1.00 (Reference) | 1.00 (Reference) |
| CRP * Females | 1.02 (0.98-1.11) | 0.241 |
| **Education** | | |
| CRP * No education | 1.00 (Reference) | 1.00 (Reference) |
| CRP * Low education | 1.04 (0.99-1.09) | 0.033 |
| CRP * Intermediate education | 0.98 (0.78-1.05) | 0.505 |
| CRP * High education | 0.83 (0.20-1.28) | 0.079 |
| **Geographical location** | | |
| CRP * Rural Ghanaians | 1.00 (Reference) | 1.00 (Reference) |
| CRP * Urban Ghanaians | 0.98 (0.95-1.01) | 0.159 |
| CRP * Amsterdam Ghanaians | 0.94 (0.74-1.06) | 0.363 |

*Results based on robust Poisson regression. Predictor =CRP *Age or sex or education or geographical location, Outcome=CKD. Male sex, no education and rural Ghanaians were reference groups. CKD is defined based on the race-free CKD-EPI 2021 equation. IRR= incidence rate ratio with 95% confidence interval.*

**Supplementary Table S4:** Association between iron status (ferritin levels) at baseline and CKD markers six years later.

| **Iron status** | **N** | **Model 1**  **IRR(95% CI)** | **Model 2**  **IRR(95% CI)** | **Model 3**  **IRR(95% CI)** | **Model 4**  **IRR(95% CI)** | **Model 5**  **IRR(95% CI)** |
| --- | --- | --- | --- | --- | --- | --- |
| **CKD incidence** | | | | | |  |
| Iron deficient | 153 | 1.03 (0.67-1.59) | 1.15 (0.65-1.92) | 1.12 (0.62-1.89) | 1.10 (0.59-1.89) | 1.11 (0.59-1.94) |
| Normal iron load | 951 | 1.00 (Reference) | 1.00 (Reference) | 1.00 (Reference) | 1.00 (Reference) | 1.00 (Reference) |
| Iron overload | 101 | 1.69 (0.99-2.71) | 1.56 (0.91-2.52) | 1.46 (0.82-2.41) | 1.38 (0.76-2.32) | 1.43 (0.67-3.09) |
| **Albuminuria** | | | | | |  |
| Iron deficient | 153 | 1.21 (0.69-1.99) | 1.24 (0.70-2.08) | 1.21 (0.67-2.07) | 1.17 (0.63-2.05) | 1.13 (0.60-2.02) |
| Normal iron load | 951 | 1.00 (Reference) | 1.00 (Reference) | 1.00 (Reference) | 1.00 (Reference) | 1.00 (Reference) |
| Iron overload | 101 | 1.46 (0.79-2.49) | 1.43 (0.78-2.44) | 1.48 (0.80-2.52) | 1.44 (0.76-2.52) | 1.64 (0.70-3.86) |
| **Decreased eGFR** | | | | | |  |
| Iron deficient | 153 | 1.01 (0.85-1.19) | 1.01 (0.83-1.19) | 0.99 (0.82-1.19) | 1.01 (0.81-1.21) | 0.99 (0.81-1.21) |
| Normal iron load | 951 | 1.00 (Reference) | 1.00 (Reference) | 1.00 (Reference) | 1.00 (Reference) | 1.00 (Reference) |
| Iron overload | 101 | 0.97 (0.78-1.20) | 0.98 (0.79-1.21) | 1.01 (0.80-1.23) | 1.01 (0.79-1.25) | 1.01 (0.74-1.35) |

*Results based on robust Poisson regression. Predictor =Iron status, Outcome=CKD or albuminuria or eGFR. CKD is defined based on the race-free CKD-EPI 2021 equation. Albuminuria, categorized as ACR < 3 and ≥3. = eGFR categorized as < 60 and ≥ 60. Iron status was based on WHO criteria where Iron deficient is ferritin <15 for both males and females; Normal is ferritin >15 to <200 in males and ferritin >15 to <150 in females: High is ferritin >200 in males and >150 in females. IRR= incidence rate ratio with 95% confidence interval.* ***Model 1****= unadjusted.* ***Model 2*** *= adjusted for age and sex.* ***Model 3*** *= model 2 + education.* ***Model 4****= Model 3 + smoking, physical activity, alcohol consumption obesity, diabetes, site, hypertension, and hypertension.* *Model 5 = Model 4 with additional adjustment for follow-up Ferritin.*

**Supplementary Table S5**: Baseline characteristics of included participants vs lost to follow-up

| **Variables** | **Included in analysis**  **N=1435** | **Lost to follow-up**  **N=2390** | **p-values** |
| --- | --- | --- | --- |
| ***Demographics*** | | | |
| Ghanaians in Rural, n (%) | 417 (29.1) | 462 (19.3) | <0.001 |
| Ghanaians in Urban, n (%) | 437 (30.5) | 840 (35.1) |  |
| Ghanaians in Amsterdam, n (%) | 581 (40.5) | 1,088 (45.5) |  |
| **Age, mean (SD)** | 46 (11) | 46 (13) | 0.8 |
| **Sex, n (%)** |  |  |  |
| Females | 899 (62.6) | 1,527 (63.9) | 0.4 |
| Males | 536 (37.4) | 863 (36.1) |  |
| **Education, n (%)** |  |  |  |
| Lower vocational | 521 (38.1) | 770 (34.7) | 0.2 |
| Intermediate | 213 (15.6) | 358 (16.1) |  |
| Higher vocational | 74 (5.4) | 119 (5.4) |  |
| **Employment status, n (%)** |  |  |  |
| Full time | 353 (32.8) | 678 (33.3) | <0.001 |
| Part-time | 578 (53.7) | 925 (45.4) |  |
| Social benefits | 51 (4.7) | 144 (7.1) |  |
| Retired | 12 (1.1) | 48 (2.4) |  |
| Unable to work | 60 (5.6) | 185 (9.1) |  |
| Student | 10 (0.9) | 40 (2.0) |  |
| ***Anthropometry information*** | | | |
| **BMI (kg/m2), median (IQR)** | 26.1 (22.4-29.9) | 26.1 (22.5-30.1) | 0.4 |
| **Waist hip ratio, median (IQR)** | 0.90 (0.86-0.95) | 0.90 (0.86-0.95) | 0.3 |
| ***Lifestyle information*** | | | |
| **Any alcohol consumption, n (%)** | 443 (37.5) | 809 (34.9) | 0.13 |
| **Smoking, n (%)** |  |  |  |
| Present | 31 (2.3) | 56 (2.6) | 0.3 |
| Past | 89 (6.6) | 173 (7.9) |  |
| **Physical activity, n (%)** |  |  |  |
| Moderate | 212 (20.5) | 354 (18.8) | <0.001 |
| High | 600 (58.0) | 984 (52.4) |  |
| ***Laboratory information*** | | | |
| **Albuminuria, n (%)** | 84 (5.9) | 329 (14.2) | <0.001 |
| **Triglycerides (mmol/L), median (IQR)** | 0.86 (0.63-1.15) | 0.86 (0.64-1.22) | 0.3 |
| **Cholesterol(mmol/L), median (IQR)** | 4.85 (4.12-5.57) | 4.83 (4.03-5.64) | 0.7 |
| **Uric acid (µmol/L), median (IQR)** | 293 (245-352) | 296 (240-361) | 0.3 |
| **Urine albumin(mg/L), median (IQR)** | 4 (4-5) | 4 (4-11) | <0.001 |
| **Urine creatinine(mmol/L), median (IQR)** | 10 (7-15) | 10 (6-16) | 0.4 |
| **Albumin creatinine ratio(mg/mmol), median(IQR)** | 0.52 (0.35-0.83) | 0.55 (0.33-1.01) | 0.015 |
| **eGFR, median (IQR)** | 87 (77-100) | 86 (74-99) | 0.001 |
| ***Underlying conditions*** | | | |
| **Hypertension, n (%)** | 542 (37.8) | 1,072 (44.9) | <0.001 |
| **Diabetes, n (%)** | 91 (6.3) | 232 (9.7) | <0.001 |
| **Obesity, n (%)** | 345 (24.1) | 608 (25.5) | 0.3 |
| ***Use of medication for the underlying health conditions*** | | | |
| **Hypertension medication, n (%)** | 204 (14.2) | 939 (39.3) | <0.001 |
| **Diabetes medication, n (%)** | 37 (2.6) | 104 (4.4) | 0.005 |
| ***Markers of inflammation*** | | | |
| **C-Reactive Protein measurement mg/L, median (IQR)** | 0.7 (0.3-2.3) | 0.7 (0.3-2.7) | 0.6 |
| **Ferritin ng/mL, median (IQR)** | 61 (29-104) | 57 (27-105) | 0.3 |

*Data are presented as percentages, means (SDs), or median (interquartile range). Percentages are rounded to one decimal point and may not sum to 100%. % = Percentages. eGFR= Estimated glomerular filtration rate. BMI=body mass index. HDL=High density lipoprotein cholesterol, LDL=Low density lipoprotein cholesterol. For p-values: Wilcoxon rank sum test; Pearson’s Chi-squared test; Fisher’s exact test was used, to compare those included and lost to follow-up.*
